# Ameliorative Effect of Areca Nut Polyphenols on Adverse Effects Induced by Lipopolysaccharides in RAW264.7 Cells

**DOI:** 10.3390/molecules29061329

**Published:** 2024-03-16

**Authors:** Luyan Zou, Shuhan Yi, Yuanliang Wang

**Affiliations:** 1 College of Food Science and Technology, Hunan Agricultural University, Changsha 410128, China; zoeluy@foxmail.com; 2 The United Graduate School of Agricultural Sciences, Kagoshima University, Kagoshima 890-0065, Japan; 3 Hunan Province Key Laboratory of Food Science and Biotechnology, Changsha 410128, China; 4 National Engineering Center of Plant Functional Components Utilization, Changsha 410128, China; 5 Changsha Innovation Institute for Food, Hunan Agricultural University, Changsha 410128, China

**Keywords:** areca nut polyphenol, RNA seq, LPS, RAW264.7

## Abstract

In Asian regions, areca nuts are tropical fruits that are extensively consumed. The areca nut contains a lot of polyphenols and its safety is unknown. In this research, we investigated the effects of lipopolysaccharides (LPS) and areca nut polyphenols (ANP) on normal RAW264.7 cells. The results showed that LPS stimulated adverse effects in normal cells by affecting cytokine production. The GO analysis results mainly affected DNA repair, cell division, and enzyme activities. In the KEGG analysis results, the NOD-like receptor signaling pathway, which is related to NF-κB, MAPK, and the pro-inflammatory cytokines, is the most significant. In the protein–protein interaction network (PPI) results, significant sub-networks in all three groups were shown to be related to cytokine–cytokine receptor interaction. Collectively, our findings showed a comprehensive understanding of LPS-induced toxicity and the protective effects of ANP by RNA sequencing.

## 1. Introduction

Areca catechu *L* is a kind of tropical fruit, which is consumed by around 600 million people worldwide, and the consumption of areca nut is increasing every year, with the main growing regions being in China, Pakistan, India, Vietnam, and so on [1,2]. Due to its capacity to eliminate parasites from the body and disperse abdominal cavity effusion, the areca nut has been used for hundreds of years as a traditional Chinese medicine and Ayurvedic medicine in India. It is regarded as one of the “four great southern medicines” in traditional Chinese medicine [3]. Nevertheless, in 2003, the International Agency for Research on Cancer (IARC) classified the areca nut as a class I carcinogen, and there are various hazards associated with the areca nut [4]. However, most of the areca nut research has been on the areca nut alkaloids and the areca nut extracts [5,6]. Few studies have been conducted on the areca nut polyphenols (ANP), and the areca nut has a high polyphenol content [7]. Polyphenols are natural compounds with great potential health value [8]. They can be used in many areas, such as food additives, disease prevention and treatment, cosmetics, and many other areas. The most common polyphenol, tea polyphenol, is a good example; tea polyphenol has antioxidants, has anti-radiation qualities, lowers blood lipids and blood sugar, and is harmless to the human body [9]. Most of the studies on betel palm polyphenols have remained at the stage of preparation and compositional analysis. A small amount of literature, indeed, shows the presence of antioxidant properties of areca nut polyphenols, which have a better ability to scavenge DPPH free radicals and ABTS free radicals, but these studies have been limited to basic phenotypic studies [10,11]. Studies on the molecular level of the antioxidant capacity of ANP and whether it also has other biological activities are almost unknown. The harmful effects of areca nut alkaloids and the unknown nature of ANP have contributed to the evaluation of the safety of the areca nut as controversial. This also makes the study of ANP all the more necessary.

The transcriptome is the sum of all the RNAs, including mRNAs and non-coding RNAs that are transcribed at a given time in a cell or a group of cells, and transcriptome research is fundamental to the study of gene function and structure. Its main purpose is to study how gene expression changes in different organisms and, thus, contributes to the understanding of human diseases [12]. It, therefore, has an important role to play in assessing, for example, drug safety and chemical risk. Because of the importance of transcriptome gene function and pathogenesis, mRNA and non-coding RNA can be detected in high-throughput sequencing and information expressed by cells at a given time can be quickly and comprehensively revealed. So, RNA sequencing will provide a comprehensive understanding of lipopolysaccharides’ (LPS) induced toxicity and the protective mechanisms of ANP, for which no studies have been reported.

In this study, the effects of ANP supplementation on transcriptome results of RAW264.7 cells were also investigated and the study provides a systematical evaluation of ANP. This is the first study to reveal the effect of ANP on RAW264.7 cells by transcriptome, offering a new direction of areca nut application.

## 2. Results

### 2.1. Summary of Data Quality

A summary of the data quality results after sequencing can be found in Table 1, in which the Q20 value indicates the percentage of bases with a base mass greater than or equal to 20 in the total number of bases, and the requirement is that the Q20 is greater than 95%, the Q30 value indicates the percentage of bases with a base mass greater than or equal to 30 in the total number of bases, and the requirement is that the Q30 is greater than 85%, and the GC value indicates the percentage of the four bases in the bases, GC, between 50 and 60%, and the requirement is that the GC is between 50 and 60%. The GC value indicates the percentage of G and C in a base out of the four bases and requires a GC between 50 and 60%. The results showed that the Q20 value of each group was greater than 96%, the Q30 value was greater than 93%, and the GC value was between 50 and 53%. Therefore, the quality of the data results is qualified and the transcriptome sequencing results have little chance of error.

### 2.2. Gene Expression Distribution and Inter-Sample Correlation

**Figure 1:**
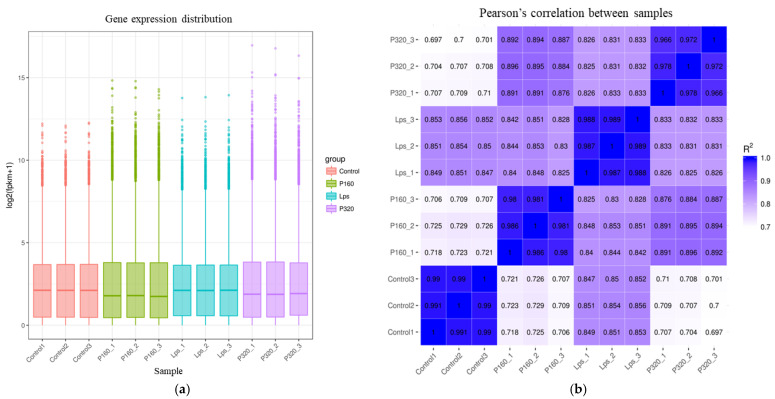
(**a**) The chart of gene expression distribution and (**b**) the heat map of sample correlation.

The distribution of gene expression levels of different samples was demonstrated by box plots, as shown in Figure 1. As can be seen from the figure, gene expression levels were similar within groups, but there were differences in gene expression between groups. Testing the experiment’s dependability and the reasonableness of the sample selection can be conducted largely by looking at the correlation of gene expression levels between samples. The results are shown in Figure 1. The correlation coefficients between groups are very small, while the coefficients within groups are large.

### 2.3. Principal Component Analysis

**Figure 2:**
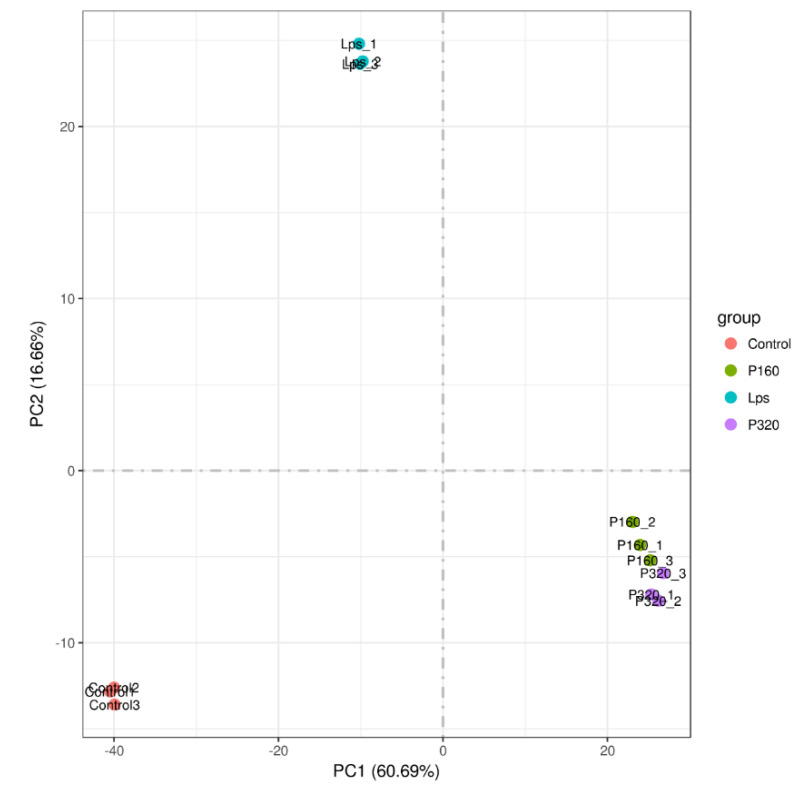
The chart of principal component analysis.

Principal component analysis (PCA) is commonly used to assess between-group differences and within-group sample repetition. The results are shown in Figure 2. The samples within the four groups are basically clustered together, and the samples among the Con, LPS, 160 μg/mL polyphenol group (P160), and 320 μg/mL polyphenol group (P320) are scattered.

### 2.4. Differential Gene Statistics

**Figure 3:**
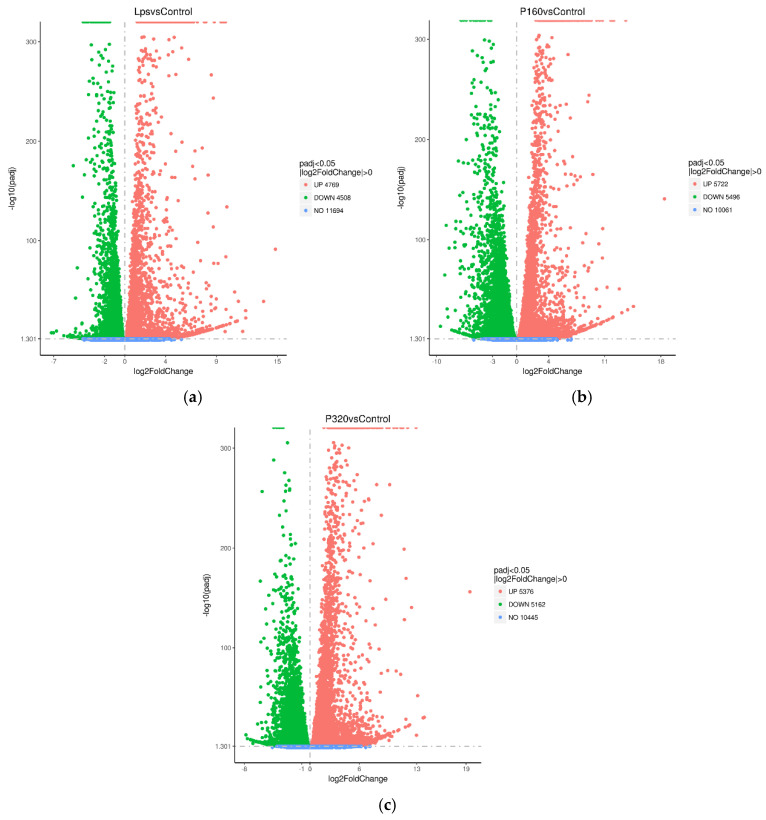
The differential gene volcano plot: (**a**) LPS/Con, (**b**) P160/Con, and (**c**) P320/Con.

All differential genes were counted and counted by the number of Log^2^ fold change. There were 9277 differential genes in LPS/Con, 11,218 differential genes in P160/Con, and 10,538 differential genes in P320/Con (Table 2). A volcano plot analysis was conducted for the gene expression between the above groups, with the horizontal coordinate indicating the fold change in expression and the vertical coordinate indicating the significance level of gene expression differences (Figure 3).

### 2.5. Gene Ontology Functional Enrichment Analysis

**Figure 4:**
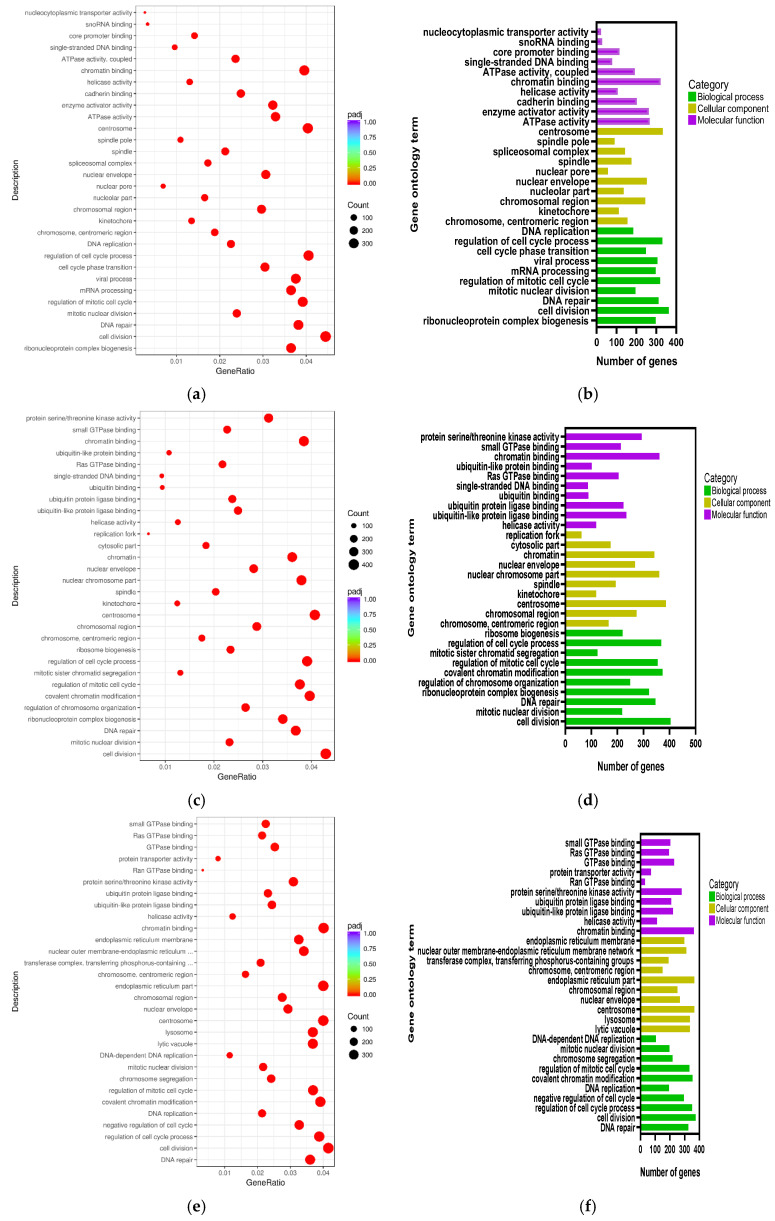
The GO ontology analysis: (**a**) LPS/Con, (**b**) LPS/Con, (**c**) P160/Con, (**d**) P160/Con, (**e**) P320/Con, and (**f**) P320/Con.

Biological process, cellular component, and molecular function are the three categories of the comprehensive GO (gene ontology) database that describes gene functions. In the LPS/Con, P160/Con, and P320/Con groups, the 10 most significant gene functions in the three sections were plotted as scatter plots and bar charts (Figure 4). The GO terms of the LPS/Con were mostly enriched in cell cycle, mitotic, and enzyme activity. The GO terms of the P160/Con were largely enriched in mitotic, enzyme activity, and binding process. The GO terms of the P320/Con were highly enriched in the cell cycle and binding process. Notably, both DNA repair and cell division appear in all three groups.

### 2.6. Kyoto Encyclopedia of Genes and Genomes Pathways Enrichment Analysis

The KEGG (Kyoto Encyclopedia of Genes and Genomes) is a comprehensive database that integrates genomic, chemical, and systemic functional information. A total of 34 pathways were enriched and significant in LPS/Con. A total of 25 pathways were enriched and significant in P160/Con. A total of 43 pathways were enriched and significant in P320/Con. Pathways associated with disease, inflammation, and cancer were counted for all three data sets. The Table 3 shows the significance of these pathways. In the LPS/Con group, 18 pathways were related to disease, inflammation, and cancer. In P160/Con, 12 pathways were enriched. In P320/Con, 18 pathways were enriched.

According to DEGs results, most of these pathways are associated with increased levels of the pro-inflammatory factor interleukin, especially IL-1 and IL-6. And the results are shown in the Table 4.

### 2.7. Protein–Protein Interaction Analysis

**Figure 5:**
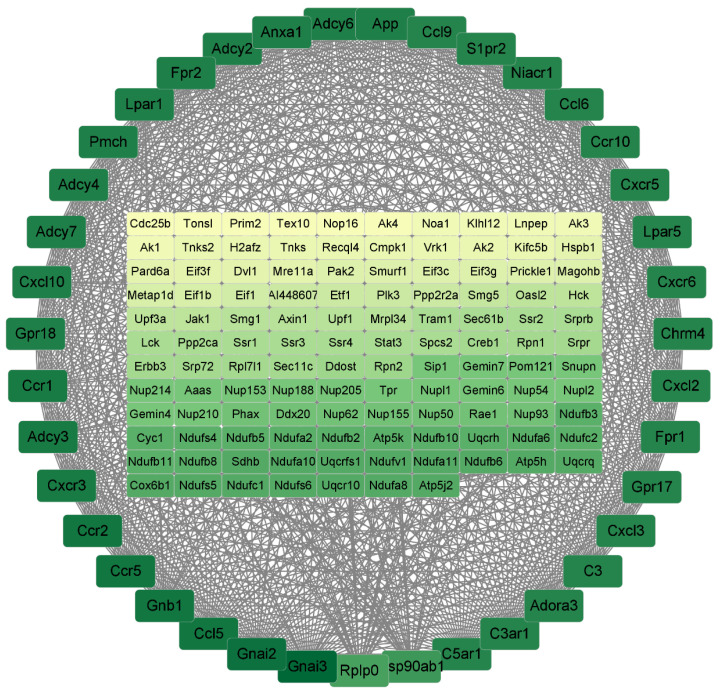
The PPI results of LPS/Con. The darker the color, the higher the degree of the gene.

Protein–protein interaction network (PPI) results were based on DEGs. The results use DEGs from LPS/Con, P160/Con, and P320/Con. The raw network was built by Cytoscape for Visualizing Molecular Interaction Networks. After building the raw network, significant sub-networks and connectivity were analyzed with MCODE and CytoNCA. For the LPS/Con group, the results are shown in Figure 5. There are a total of 156 genes in it, arranged in order and color according to degree; the higher the degree, the darker the color. The degrees of the top 10 genes are shown in Table 5 below. All of them are associated with cytokine–cytokine receptor interaction.

**Figure 6:**
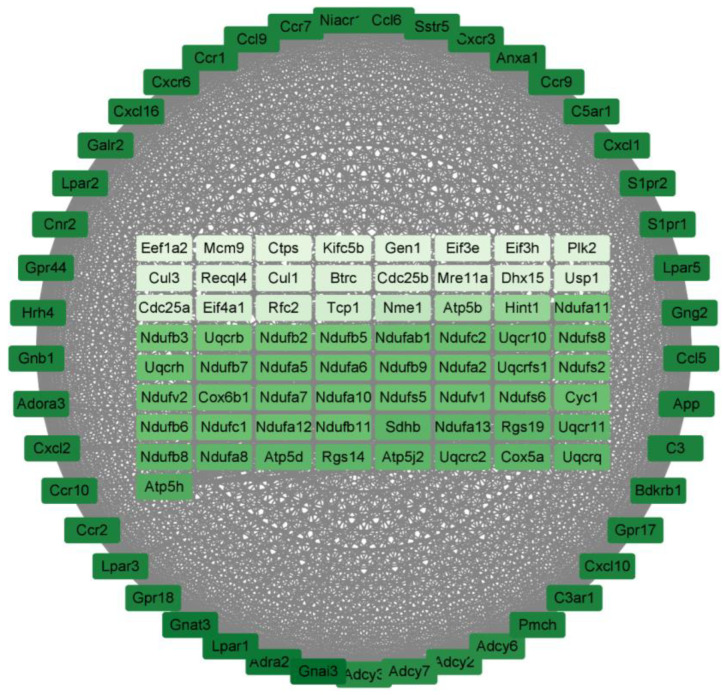
The PPI results of P160/Con. The darker the color, the higher the degree of the gene.

For the P160/LPS group, the results are shown in Figure 6. There are a total of 110 genes in it, arranged in order and color according to degree; the darker the color, the higher the degree. The degrees of the top 10 genes are shown in Table 6 below. All of them are associated with cytokine–cytokine receptor interaction.

**Figure 7:**
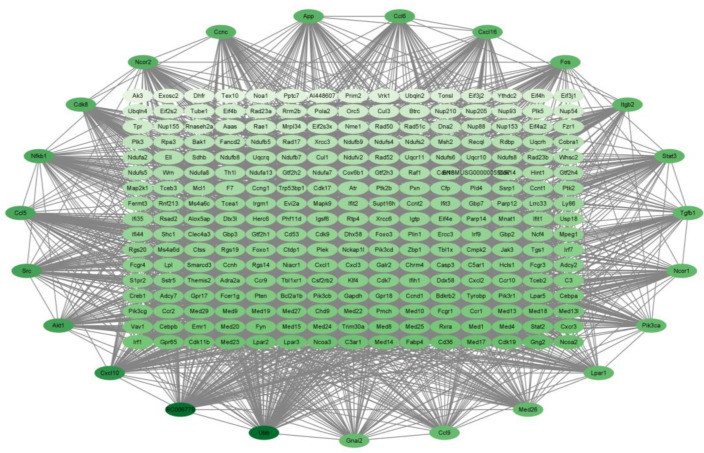
The PPI results of P320/Con. The darker the color, the higher the degree of the gene.

For the P320/LPS group, the results are shown in Figure 7. There are a total of 278 genes in it, arranged in order and color according to degree; the darker the color, the higher the degree. The degrees of the top 10 genes are shown in Table 7 below. All of them are associated with cytokine–cytokine receptor interaction.

## 3. Discussion

The IARC has classified the tropical fruit areca nut as a class 1 carcinogen because of its detrimental impact on oral health. However, it has been used for thousands of years in traditional Chinese and Indian medicine, and the areca nut has anthelmintic properties [13]. This may be because the areca nut is rich in polyphenols [14]. LPS is an endotoxin whose physiological role is manifested through Toll-like receptor 4 present on the surface of the cell membrane of the host cell [15]. It is mostly used to stimulate cells to produce inflammation. ANP has antioxidant and anti-inflammatory effects; it has been demonstrated in previous studies that ANP attenuates cellular oxidative stress and inflammation induced by LPS through the Nrf2/HO1 and MAPK pathways [16].

In the results of transcriptomics, differential gene expression varied considerably between groups. In the results of the correlation analysis, it can be seen that there are differences between all groups, but the correlation between P160 and P320 is close. This indicates that the expressed differential genes in the two groups are very similar. In the PCA results, it can be seen that Con, LPS, and the sample treatment groups are dispersed. However, within the groups, they are clustered together. This further proves that there is a large difference in gene expression between the groups.

Among the GO results, our findings in the three groups mainly affect DNA repair, cell division, enzyme activity, and binding processes. Based on the KEGG results, there are 18 disease-related pathways in the Con/LPS group after LPS stimulation. In P160/Con, there were 9 fewer disease pathways compared to the LPS/Con group, but there were also 3 new pathways. In 320/Con, there were 7 fewer disease pathways compared to the LPS/Con group, but there were also 7 new pathways. Among them, the NOD-like receptor signaling pathway is the most significant. The NOD-like receptor signaling pathway triggers the generation of pro-inflammatory cytokines and activates NF-κB and MAPK in response to different infections [17,18]. This suggests that LPS is activating MAPK and NF-κB via the NOD-like receptor signaling pathway to produce pro-inflammatory factors, and this result is consistent with previous studies [19]. It is also shown in the DEGs results that IL-1- and IL-6-related genes are also reduced in P160/Con and P320/Con compared to LPS/Con, and, in combination with our previous studies, it is also known that LPS increases the expression of the MAPK pathway and that areca nut polyphenols attenuate this expression [16]. This suggests that ANP also ameliorates the stimulatory effects of LPS by affecting the NOD-like receptor signaling pathway, thereby reducing the production of pro-inflammatory factors.

Protein–protein interactions are important in predicting target protein function and compound drug ability [20] through analyzing the important sub-networks in the PPI network. In the sub-network result, it was learned that all of the three groups affect cytokine–cytokine receptor interaction. And the degree of the genes related to cytokines in them are all high. Notably, the Gnai3 gene had the highest degrees in both the LPS/Con and P160/Con groups. The Gnai3 gene is a gene closely related to pro-inflammatory factors and diseases [21,22]. This implies that the adverse effects of LPS on normal cells are produced by stimulating cytokine production, which is consistent with other LPS studies [23].

## 4. Materials and Methods

### 4.1. Preparation of Areca Nut Polyphenols

Areca catechu raw material was obtained from Wanning City, Hainan, China, in January 2018. ANP was extracted as described. Essentially, areca nut fruits were freeze-dried and crushed into powder. After 48 min at 68 °C and 50% (*v*/*v*) ethanol extraction, it was freeze-dried. A 50% ethanol wash was performed after crude polyphenols were refined using XAD-7 macroporous resin. The total polyphenol content was determined to be 80% using the Folin–Ciocalteu approach [24]. Previous research found that areca nuts contain two types of polyphenols: catechins (2060.44 ± 18.24 g/mL) and proanthocyanidin B1 (2510.18 ± 62.40 g/mL) [25].

### 4.2. Cell Culture

The American Type Culture Collection (Manassas, VA, USA) provided the RAW264.7 macrophages, which were then cultured in DMEM containing 10% FBS and 1% antibiotic at 37 °C in 5% CO_2_ humidified air. The cells were cultivated in 96-well plates and the number of cells per well was 2 × 10^4^. After 24 h of culture, five different concentrations (40, 80, 160, 320, and 640 μg/mL) and four treatment times (6, 9, 12, and 15 h) were set. According to the MTT and antioxidant results of the previous study, we used the 160 and 320 μg/mL for the next experiments [16].

### 4.3. RNA Extraction and Quality Control

On 1% agarose gels, RNA contamination and degradation were observed. The nanophotometer spectrophotometer (IMPLEN, Westlake Village, CA, USA) was used to verify the purity of the RNA. Using the Qubit^®^ RNA assay kit in the Qubit 2.0 fluorometer (Life Technologies, Carlsbad, CA, USA), the concentration of RNA was determined. The RNA Nano 6000 assay kit (Agilent Technologies, Santa Clara, CA, USA) for the Bioanalyzer 2100 system (Agilent Technologies, Santa Clara, CA, USA) was used to assess the RNA’s integrity.

### 4.4. Library Preparation for Transcriptome Sequencing

The RNA sample preparations employed 3 μg of RNA total per sample as input material. According to the manufacturer’s instructions, sequencing libraries were created using the NEBNext UltraTM RNA library prep kit for Illumina^®^ (NEB, Ipswich, MA, USA), and index codes were added to each sample to identify its sequences. To put it briefly, mRNA and total RNA were separated using magnetic beads coupled to poly-T oligo. Fragmentation was performed at high temperatures in NEBNext first strand synthesis reaction buffer (5×) using divalent cations. M-MuLV reverse transcriptase (RNase H-) and a random hexamer primer were used to create the first strand of cDNA. RNase H and DNA polymerase I were then used to synthesize the second strand of cDNA. The remaining overhangs were transformed into blunt ends using polymerase and exonuclease reactions. To become ready for hybridization, NEBNext adaptors with hairpin loop structures were ligated after the 3’ ends of DNA fragments had been adenylated. The AMPure XP system (Beckman Coulter, Beverly, CA, USA) was used to purify the library fragments to choose cDNA fragments that were preferably 250–300 bp in length. Subsequently, 3 μL USER enzyme (NEB, USA) was employed with adaptor-ligated, size-selected cDNA at 37 °C for 15 min, and then 5 min at 95 °C before PCR. After that, Phusion high-fidelity DNA polymerase, universal PCR primers, and index (X) primer were used to carry out PCR. Finally, the AMPure XP system was used to purify the PCR products and the Agilent bioanalyzer 2100 system was used to evaluate the quality of the libraries.

### 4.5. Clustering and Sequencing (Novogene Experimental Department)

The index-coded samples were clustered using a cBot cluster generation system with the TruSeq PE cluster kit v3-cBot-HS (Illumia, Bologna, Italy) according to the manufacturer’s instructions. Following cluster formation, 125 bp/150 bp paired-end reads were produced by sequencing the library preparations on an Illumina Hiseq platform.

### 4.6. GO and KEGG Enrichment Analysis of Differentially Expressed Genes

The cluster profiler R program was used to perform a gene ontology (GO) enrichment analysis of differentially expressed genes, after correcting for gene length bias. Genes that were differentially expressed were thought to significantly enrich GO keywords if the adjusted *p*-value was less than 0.05. Gene-level functions and utility of biological systems, such as cells, organisms, and ecosystems, are provided by the KEGG database, which is derived from molecular-level data, particularly from large molecular datasets generated by high-throughput experimental methods like genome sequencing. We used the cluster profiler R program (v3.5.1) to investigate the statistical enrichment of differential expression genes in KEGG pathways.

### 4.7. PPI Analysis of Differentially Expressed Genes

The STRING database, which identifies and predicts protein–protein interactions, served as the foundation for the PPI analysis of genes with differential expression. The total network of each group was plotted using Cytoscape (v3.9.1) based on the DEGs data. Significant sub-networks in the total network were analyzed by using MCODE and CytoNCA, and the degree of each differential gene in the sub-network was calculated.

### 4.8. Data Analysis

PRISM was used to depict all charts and one-way ANOVA (SPSS23, 2010) was used to analyze all data and least-significant difference (LSD) and Duncan’s multiple range tests were used to assess the differences between treatment groups, with a significance level of *p* < 0.05 or *p* < 0.01 [26].

## 5. Conclusions

In conclusion, in the quantitative analysis, we can see that there are differences in the genes between the groups. Then, using GO and KEGG analysis, we studied the effects of LPS through pro-inflammatory factors and activation of inflammatory pathways and, thus, adverse effects, which were improved after ANP treatment. In the PPI results, it was also shown that LPS stimulated cytokine production and ANP reduced IL-1- and IL-6-related genes. Our research efforts provide a comprehensive understanding of LPS-induced toxicity and the protective effects of ANP by RNA sequencing and this provides theoretical support for the safety of areca nut polyphenols and provides new ideas for future research on the safety and application of the areca nut.

## Figures and Tables

**Table 1:** The table of data quality summary.

Sample	Q20 %	Q30 %	GC %
Con-1	97.81	93.85	50.37
Con-2	97.65	93.48	50.58
Con-3	97.62	93.43	50.45
LPS-1	97.88	94.02	50.45
LPS-2	97.9	94.04	50.65
LPS-3	97.78	93.77	50.51
160-1	97.88	94.17	52.08
160-2	97.85	94.09	51.77
160-3	97.73	93.83	52.81
320-1	97.73	93.8	52.35
320-2	97.63	93.55	52.57
320-3	97.53	93.4	52.68

**Table 2:** The statistical table of differential genes.

Log^2^ Fold Change	LPS/Con	P160/Con	P320/Con
≥10	14	26	26
≤5 to <10	282	540	629
≤2 to <5	847	1552	1804
<−2 to <2	7699	7104	6654
<−5 to ≤−2	419	1821	1350
<−10 to ≤−5	16	175	75
≤−10	0	0	0

**Table 3:** The KEGG results of PLPS/Con, P160/Con, and P320/Con.

Pathway	LPS/Con	P160/Con	P320/Con
NOD-like receptor signaling pathway	0.001569	0.00867	0.015443
Tuberculosis	0.004478	0.022062	0.021961
TNF signaling pathway	0.00479	0.000416	0.004654
Toll-like receptor signaling pathway	0.00594	0.038412	0.000644
Ferroptosis	0.009903	-	-
Fanconi anemia pathway	0.016513	0.038412	0.010493
Hepatitis B	0.01739	0.024212	0.000698
Influenza A	0.01739	-	0.022635
Cytosolic DNA-sensing pathway	0.021311	-	-
Acute myeloid leukemia	0.02167	-	0.022635
Non-small cell lung cancer	0.02167	-	0.021167
Chronic myeloid leukemia	0.040914	-	0.02252
Alzheimer’s disease	0.045233	-	-
Huntington’s disease	0.045233	0.043236	-
Necroptosis	0.045233	0.035543	-
NF-kappa B signaling pathway	0.045233	0.024977	0.018269
Glioma	0.045233	-	-
p53 signaling pathway	0.045934	-	-
Herpes simplex infection	-	0.015792	0.033036
Non-alcoholic fatty liver disease (NAFLD)	-	0.024212	-
Measles		0.029613	-
Chagas disease (American trypanosomiasis)			0.004859
Kaposi’s sarcoma-associated herpesvirus infection			0.007525
Cellular senescence			0.014608
Epstein–Barr virus infection			0.031664
Leishmaniasis			0.03858
Small cell lung cancer			0.043946

**Table 4:** The DEGs in LPS/Con, 160/Con, and 320/Con.

Gene Name	Log^2^ Fold Change		
	LPS/Con	P160/Con	P320/Con
IL-1α	8.025507	4.466649	4.1412
IL-1β	10.79287	4.484613	4.872983
IL-18	2.84762	1.954871	1.04118
IL-6	10.34082	10.55272	8.590223
IL-11	4.788148	5.663995	5.67686

**Table 5:** The 10 hub genes with the highest level of connectivity in LPS/Con.

Gene Symbol	Gene Description	Degree
Gnai3	G protein subunit alpha i3	42
Gnai2	G protein subunit alpha i2	40
Ccl5	C-C motif chemokine 5	39
Gnb1	Guanine nucleotide-binding protein subunit beta-1	39
Ccr5	C-C chemokine receptor type 5	39
Ccr2	C-C chemokine receptor type 2	39
Cxcr3	C-X-C chemokine receptor type 3	38
Adcy3	Adenylate cyclase 3	38
Ccr1	C-C chemokine receptor type 1	38
Gpr18	N-arachidonyl glycine receptor	37

**Table 6:** The 10 hub genes with the highest level of connectivity in P160/Con.

Gene Symbol	Gene Description	Degree
Gnai3	G protein subunit alpha i3	48
Adra2a	Adrenergic receptor alpha-2A	47
Lpar1	Lysophosphatidic acid receptor 1	47
Gnat3	Guanine nucleotide-binding protein G(t) subunit alpha 3	47
Gpar18	N-arachidonyl glycine receptor	46
Lpar3	Lysophosphatidic acid receptor 3	46
Ccr2	C-C chemokine receptor type 2	46
Ccr10	C-C chemokine receptor type 10	46
Cxcl2	C-X-C motif chemokine ligand 2	46
Adora3	Adenosine receptor A3	46

**Table 7:** The 10 hub genes with the highest level of connectivity in P320/Con.

Gene Symbol	Gene Description	Degree
Ubb	Ubiquitin B	89
BC006779	—	88
Cxcl10	C-X-C motif chemokine 10	71
Akt1	AKT serine/threonine kinase 1	63
Src	Tyrosine-protein kinase Src	63
Ccl5	C-C motif chemokine 5	61
Nfkb1	Nuclear factor NF-kappa-B p105 subunit	60
Cdk8	Cyclin-dependent kinase 8/11	56
Ncor2	Nuclear receptor co-repressor 2	56
Ccnc	Cyclin-C	55

## Data Availability

All relevant data are presented in this study.
